# p53 dependence of senescence markers p21v1 and p21v2 in aging and acute injury

**DOI:** 10.1038/s41514-024-00175-z

**Published:** 2024-10-14

**Authors:** Parmita Kar, Ashok Sivasailam, Rupa Lavarti, Lun Cai, Muthusamy Thangaraju, Emma Nguyen, Bhavishya Mundluru, Raghavan Pillai Raju

**Affiliations:** 1 Department of Pharmacology and Toxicology, Medical College of Georgia, Augusta University, Augusta, GA USA; 2 Department of Biochemistry and Molecular Biology, Medical College of Georgia, Augusta University, Augusta, GA USA

**Keywords:** Pathogenesis, Biomarkers

## Abstract

The senescence phenotype is heterogeneous, as observed by the context-dependent differential expression of senescence markers. Here, we provide evidence to demonstrate an inverse relationship in the expression pattern of the two murine variants of *p21* (*p21v1*, and *p21v2*) in aging and hemorrhagic shock. While an upregulation of *p21v1* was observed following hemorrhagic shock injury, *p21v2* was upregulated in the aged mouse. We further show that the *p21v1* response is, at least, partially independent of *p53*.

Cellular senescence is a state of cell cycle arrest in which cells irreversibly cease cell division, resist apoptosis, and contribute to the pro-inflammatory environment^1–3^. In addition, senescence may also be viewed as a homeostatic necessity in processes such as embryonic development and tissue repair^3^. Studies have demonstrated cellular senescence in acute lung injury, including fibrosis, acute kidney injury, acute pancreatitis, and endothelial dysfunction^4–10^. While targeted removal of senescent cells by senolytics was shown to have a therapeutically effective role in age-associated comorbidities, a recent report from our lab demonstrated a rapid senescence-like response in rat liver following hemorrhagic shock injury (HI) and suggested a protective role for senescence in this acute injury^11,12–14^.

The cyclin-dependent kinase inhibitors, *Cdkn1a* (also known as p21 or CIP1 or WAF1) and *Cdkn2a* (also known as p16INK4A), are classic markers of senescent cells that drive the senescence programs^15^. While p21 participates in the activation and initiation of senescence, some studies describe p16 as a late-stage marker^16^. The tissue-specific expression of p16 and p21 with age in humans and mice have been investigated using different techniques and tools such as immunohistochemistry, progeroid mice, and in-silico mining of RNA-seq data^17–22^. In mice, p21 is known to generate at least two transcript variants due to alternative transcription start sites leading to different 5’ untranslated regions^23,24^. However, there is a knowledge-based gap in the understanding of the tissue-cell-type specific expression patterns, role, and regulation of the p21 transcript variants in stress or age-associated senescence. A recent report identified the expression of p21 variants 1 and 2 (p21v1 and p21v2) in murine cells exposed to genotoxic stress and demonstrated a rapid expression of variant 1 compared to that of variant 2^24^.

In this report, we address the context-specific differential expression of p16, p21, and the p21 variants in different organs in a mouse model of HI (acute injury model) compared to healthy young and aged mice. We report an inverse relationship between aging and hemorrhagic shock in the expression pattern of p21 v1 and v2 variants. Furthermore, this work, for the first time, provides important experimental evidence for the p53-dependent regulation of p21 variants.

Our experiments demonstrated a significantly higher induction of p21 gene expression in the liver, lung, heart and kidney in the aged and all the organs tested in mice subjected to HI (Fig. 1a–d and Supplementary Fig. S1). In contrast, p16 expression was not increased following HI in any of the organs tested. However, tissues from aged animals demonstrated a significant increase in p16 gene expression. On average, the relative abundance of p21 was more than 4-fold in lung, liver, heart and kidney, respectively, in the aged mice, compared to the young mice. Additionally, the p21 levels increased approximately 30-fold in the heart and liver and 16-fold in the lung and kidney following HI. Similarly, p16 showed about a 4-fold increase in aged lung and heart and more than 15-fold in liver and kidney, respectively.

**Fig. 1:**
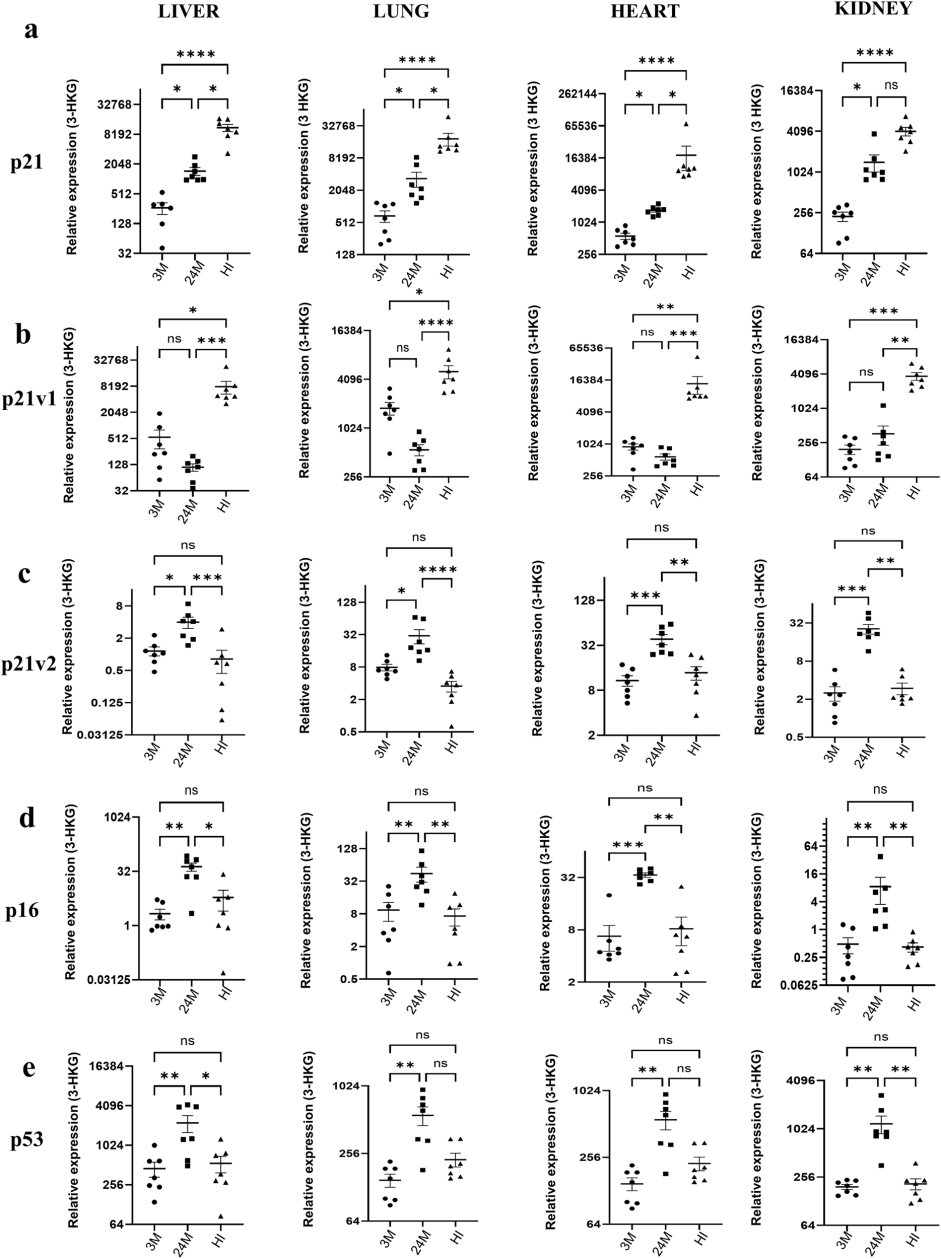
The cdkn1a (p21) and 2a (p16) are differentially regulated in aging and HI. **a**–**e** mRNA levels of p21, p21 variant 1, p21 variant 2, p16 and p53 in the liver, lung, heart and kidney of male mice aged 3 months (3M, young) and 22–24 months (24M, aged), and male mice subjected to hemorrhagic shock injury (HI, young mice). Seven animals per group and at least two technical replicates used. The data was normalized to the geometric mean of three housekeeping genes (HKG): β-actin, β-glucuronidase and Rplp0 (ribosomal protein large P0). The graphs are shown as individual data points along with mean ± SEM. The *Y*-axes are in log_2_ scales. Kruskal–Wallis non-parametric test was used. *p < 0.05, **p < 0.01, ***p < 0.001, ****p < 0.0001; ns = not-significant.

As p21 was found to be upregulated in both aging and the acute injury model, to determine whether there is a difference in the expression of p21 transcript variants in these conditions, we tested the gene expression of p21 variants v1 and v2 (Fig. 1b, c, and Supplementary Fig. S1). When compared to the expression in healthy young mice, only the p21v1, not p21v2, was found to significantly increase in the tissues in mice subjected to hemorrhagic shock. An inverse expression pattern was observed in aged mice where variant 2 was significantly upregulated, not variant 1.

To confirm whether the differential expression of p21 and p16 proteins follows the gene expression pattern we observed, we tested liver tissues from mice subjected to HI or sham surgery and WT aged mice by immunofluorescence. We observed an elevated expression of p21 (green, Fig. 2b) in the liver of mice subjected to HI compared to the sham. In contrast, in the aged mice, both p16 and p21 (red and green, respectively) showed increased expression and no significant p16 staining was found in the tissues from the HI mice (Fig. 2a, b). We observed a nucleo-cytoplasmic localization of p21 in liver tissues from aged as well as HI mice. Western blot of tissues from young, aged and HI using p21 antibody also showed a similar trend of protein expression as observed in RT-PCR experiments (Supplementary Fig. S3). The p21 staining in the liver section of aged and HI mice was mostly in the hepatocytes (Fig. 2c).

**Fig. 2:**
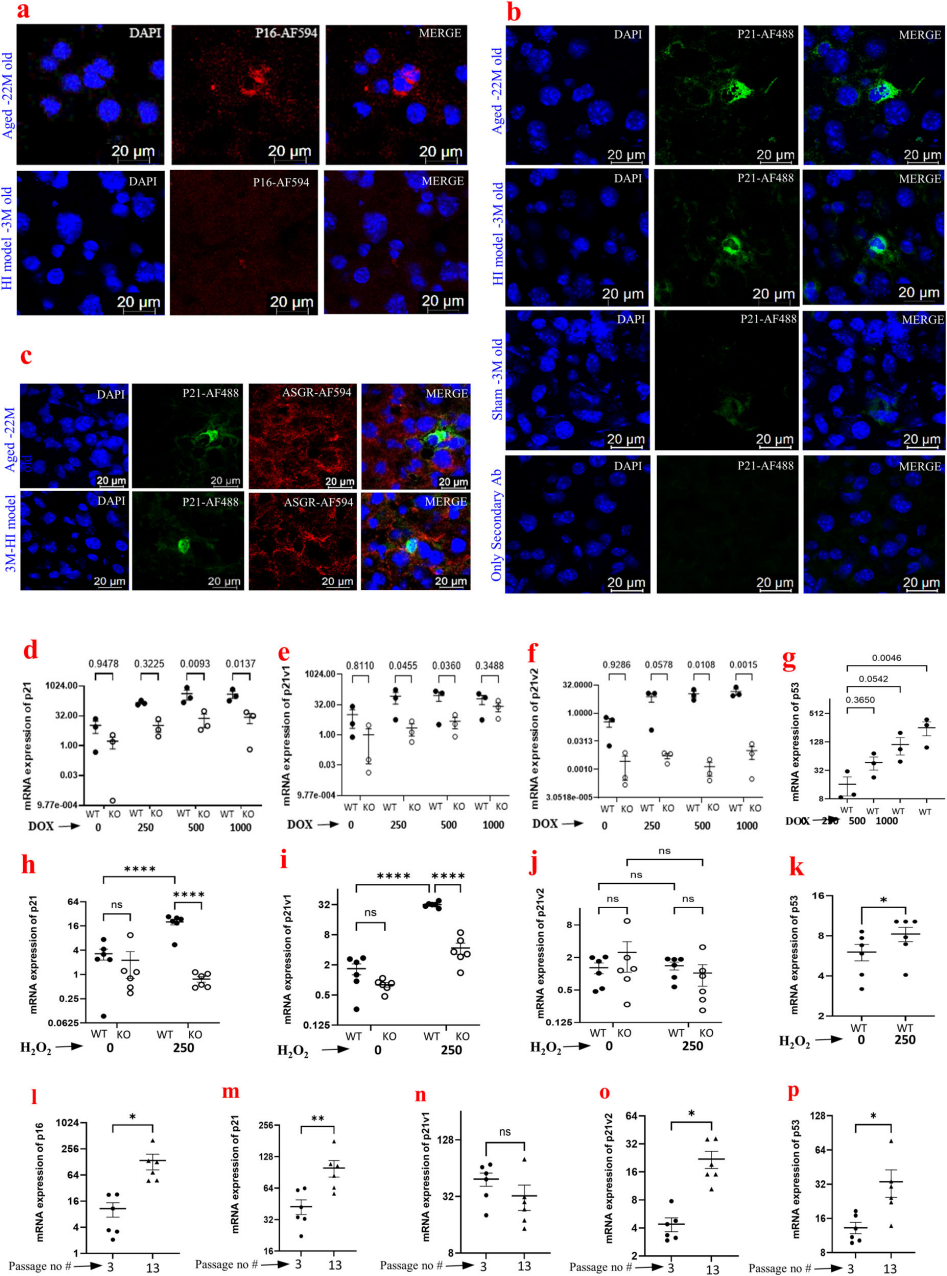
Characterization of senescence markers p21 and p16 in aging and HI. **a** Immunolocalization of p16 (red) in liver tissues of 3-month-old C57/BL6 mice subjected to hemorrhagic shock compared to aged C57BL/6 (22-month-old) mice. **b** Immunolocalization of p21 (green) in young-HI mice compared to young sham-operated mice and aged mice. No primary antibody was used as negative control. **c** Liver sections from aged and HI mice stained with antibodies to p21 (green), hepatocyte marker ASGR (red), and DAPI (cyan/blue) showing co-localization of p21 to hepatocytes. Nucleus was stained using DAPI (blue). All the images were captured at a total magnification of 630×. **d**–**f** mRNA expression of p21, p21v1 and p21v2 in MEF cells from p53-KO and WT littermate mice and **g** p53 in WT-MEF cultured and treated with various concentrations of doxorubicin for 48 h. The experiment had three biological replicates and the PCR was repeated twice. **h**–**j** mRNA expression of p21, p21v1 and p21v2 in MEF cells from p53-KO and WT littermate mice and **k** p53 in WT-MEF cultured and treated with 250 µM H_2_O_2_ for 24 h. **l**–**p** mRNA expression of p16, p21, p21v1 and p21v2 in MEF cells at late passage 13 in comparison to early passage 3. The experiments (**h**–**p**) had six biological replicates and the PCR was repeated twice. The data was normalized to β-actin in all experiments. The *Y*-axes are log_2_ scales. Results were analyzed using Kruskal–Wallis (**d**–**g**) 2-way ANOVA (Uncorrected Fisher’s LSD) (**h**–**j**), Wilcoxon’s (**k**) and Mann–Whitney (**l**–**p**) test. *p* < 0.05 was considered significant (*p < 0.05, **p < 0.01, **p < 0.001, ***p < 0.0001), unless absolute *p*-value indicated.

The expression of p21 is regulated by p53. However, the role of p53 in the differential regulation of p21v1 and p21v2 is unclear. As shown in Fig. 1e, p53 was significantly upregulated in all the aged tissues. On the contrary, the p53 levels did not show any significant change in the tissues harvested from mice subjected to HI. Therefore, to understand the p53-dependent regulation of the p21 transcript variants, we conducted additional experiments using p53^−^^/^^−^ MEF cells.

Doxorubicin is known to induce p53 and p21. Doxorubicin at concentrations 250nM-1μM induced a significant increase in the expression of p21 in wild-type MEF (WT-MEF) cells (Fig. 2d). However, the expression levels of p21 were several-fold lower in doxorubicin-treated p53^−^^/^^−^-MEF than in WT-MEF cells. The p21v1 expression was upregulated at higher concentrations of doxorubicin in p53^−^^/^^−^ cells, though the levels were still lower than in WT cells. Interestingly, p21v2 expression, though increased in WT cells in response to doxorubicin treatment, was barely detectable in the p53^−^^/^^−^ MEF cells demonstrating p53 dependence for the p21v2 variant expression (Fig. 2e, f).

Though the senescence induced by doxorubicin treatment may not reflect the physiological response to hemorrhagic shock or aging, the upregulation of both p21 v1 and v2 in this model allowed us to test the role of p53 in regulating their differential expression. We also treated MEF cells with H_2_O_2_, an agent that is known to induce oxidative stress. Oxidative stress is a common denominator between aging and hemorrhagic shock^25^. We investigated the effect of p53 on v1 and v2 expression in the in vitro model of H_2_O_2_-induced oxidative stress (Fig. 2h–j). While both v1 and v2 were found to increase with doxorubicin in WT cells, only v1 was found to be elevated in response to H_2_O_2_ in the WT-MEF. Furthermore, the p53 expression was significantly increased in WT-MEF cells after H_2_O_2_ treatment similar to that observed in response to doxorubicin treatment (Fig. 2g, k). We also observed that MEF cells passaged to senescence upregulated p16, p21, p21v2 and p53, but not p21v1, unlike that in the H_2_O_2_-treated cells (Fig. 2l–p, Supplementary Fig. S2).

Senescent cells exhibit molecular and functional heterogeneity depending on cell types and physiological contexts^26^. Tissue-specific variations in secretome profiles of p21 and p16 expressing cells were demonstrated with scRNA-seq datasets in murine and aged human tissues, suggesting distinct functional roles of these subpopulations^19^. We examined the expression changes of the two major molecular markers of senescence, p16 and p21, and the two known murine p21 variants following acute injury using our HI model. Our study confirms that while the tissues from aged animals show the upregulation of both p16 and p21, acute injury-related senescence shows the upregulation of only p21. This dichotomy may be because aging is associated with both replicative and stress-induced senescence, and/or because aging is a slow process compared to acute injuries. It is also pertinent to note that, as we have reported earlier, the senescence phenotype emerges in animals subjected to HI rapidly, within hours, though its functional relevance remains unknown^11^.

Our study shows that the protein expression of the senescence markers follows a similar pattern as gene expression. Interestingly, the immunofluorescence studies show a nucleo-cytoplasmic localization of p21 in HI and aged liver tissues. Previous reports suggest that phosphorylation at Thr-145 and Ser-146 targets p21 to the cytoplasm^27^ and that delayed apoptosis in cells expressing the phosphorylated form of p21 may promote tumorigenesis^28,29^.

A recent study by the Campisi group suggested that senescence may be characterized by differential expression of gene variants of p21, depending on the senescence inducer, with variant 1 responsive to stress-induced senescence and variant 2 to age-associated senescence^18^. This is consistent with our results demonstrating an upregulation of p21v2 in aged tissues in contrast to the upregulation of p21v1 in hemorrhagic shock injury (HI). The p21 variants 1 and 2 mRNAs differ only in their 5’ untranslated regions (UTR) but translate identical proteins^30^. The variant v2 has a longer 5’ UTR and contains 5’ upstream ORFs. This difference in UTRs may also be attributed to the differential transcription of p21 variants due to alternative promoter usage, depending on the nature of the stress^23^.

Though p21 was originally described as a p53 target, some stress factors, such as Ras oncogene, have been shown to upregulate p21 independently of p53^31^. The p65 (also called RelA) subunit of NF-kB can also mediate the regulation of p21 in the absence of p53^32^. However, the role of p53 in differential regulation of p21 variants remains unclear. Our results show that p53 is upregulated in tissues from aged mice but not HI mice. In our previous report on HI-induced senescence in rat liver, we observed that total p53 protein level remained unchanged after HI, but phosphorylated p53 was increased^11^. The variation in expression of p53 with respect to upregulation of variant 1 in injury and variant 2 in aging is likely due to the proximity of p21v2-TSS to the p53 response elements^24^.

Our next experiment was to test whether there is a p53-dependent or independent upregulation of any of the p21 variants. Our experiments, for the first time, provide experimental evidence that the absence of p53 leads to significantly diminished expression of p21v2 but not p21v1, in murine MEF cells in response to doxorubicin, a classical inducer of senescence. Conversely, an in vitro model of H_2_O_2_-induced oxidative stress showed a lack of p21v2 induction in both WT and p53-deficient MEF. Furthermore, while p21v1 and v2 were upregulated in the WT cells treated with doxorubicin, only v1 was significantly upregulated with H_2_O_2_ in WT-MEF, as observed in HI.

In summary, we demonstrate differing senescence phenotypes in response to aging, HI, and in vitro stress models in terms of expression of p16, p21, and p21 variants and their response to p53. Further studies are required to address the unmet need to comprehensively assess the heterogeneity of p21 and its variants in cellular senescence at single-cell level in a tissue-specific manner. The post-transcriptional regulation of p21 and its variants by p53 and other factors under various conditions of stress need further detailed investigations, to further the biology of aging and senescence as well as the outcome trajectory of acute injuries.

## Materials and methods

### Ethics approval statement

The animal use and experiments including hemorrhagic shock injury (HI) described in the study were approved by the Institutional Animal Care and Use Committee (IACUC) at Augusta University and were performed in accordance with the relevant guidelines and regulations.

### Animals

The young (3 months old) and old (22–24 months old) C57BL/6 male mice used were either bred in-house or purchased from the National Institute on Aging. All the mice were housed in the vivarium at the Augusta University with a 12 h light/ dark cycle.

### Hemorrhagic shock injury procedure

The animals were subjected to sham or HI procedure as described earlier^33,34^. Briefly, the animals were anesthetized with isoflurane (Henry Schein, Dublin, OH, USA). Both the femoral arteries were cannulated for bleeding, blood pressure monitoring, or fluid resuscitation. Sham animals did not undergo hemorrhage or fluid resuscitation. HI was induced by bleeding rapidly to a MAP of 35 ± 5 mmHg in 45 min by removing ~60% of the total blood volume. The animals were then maintained in a state of shock by maintaining the low MAP for another 45 min, after which fluid resuscitation was carried out with Ringer lactate (RL; twice the volume of shed blood in 1 h). The animals were euthanized at the end of 2 h post-fluid resuscitation, the heart was perfused with ice-cold PBS, and the tissues were collected.

### RNA extraction and PCR

RNA was extracted from young, aged and HI tissues using Trizol-reagent (Invitrogen, CA, USA). The RNA quality was ascertained by A260/280 as well as by resolving on 2% agarose gel to assess the 28S and 18S bands. cDNA synthesis was carried out using ImProm-II reverse transcriptase (Promega, WI, USA). The transcripts were analyzed using Bio-rad iTaq Universal SYBR Green Master mix (Bio-rad, CA, USA) in an Agilent real-time PCR machine (Agilent Technologies, CA, USA). The PCR results were normalized to the geometric mean of *β-actin, β-glucuronidase* and *ribosomal protein large P0* (*Rplp0*)^35–37^. Primer sequences are listed in Supplementary Table 1. 2^ΔCt^ values were used for statistical analyses, and the graphs are represented in log_2_ scale.

### Cell culture and induction of senescence

p53^−^^/^^−^ and wild-type littermate MEF (WT-MEF) cells were treated with 250 nM–1μM concentrations of doxorubicin for 48 h and harvested for molecular studies. Additionally, an H_2_O_2_-induced stress model was used by treating p53^−^^/^^−^ and wild-type littermate MEF (WT-MEF) cells with 250 μM concentration of H_2_O_2_ for 24 h. To examine the expression of senescence markers in replicative senescence, MEF cells from passage three (P3, denoted as early passage) were serially passaged after 80% confluence until passage 13 (P13, denoted as late passage). Cells from early as well as late passage were then used to check the expression of senescence markers p16, p21 and p53.

### Indirect immunofluorescence and confocal microscopy

Protein expression of p16 and p21 was assessed by indirect immunofluorescence. Briefly, the cryo-sections were blocked using 2% BSA for 1 h and a cocktail of p21 (1:150, Cat. No sc-6246, Santa Cruz Biotechnology, CA, USA) and p16 (1:100, Cat. No. PA5-20379, Invitrogen, CA, USA) unconjugated primary antibodies were used. The slides were then washed with PBS with and without 0.075% Brij-35 detergent (Thermo Fisher Scientific) and incubated with a cocktail of secondary anti-mouse 488 for p21 (1:1500, Abcam, MA, USA), anti-rabbit 594 for P16 (1:1500, Invitrogen) for 2 h. The slides were again washed and incubated with DAPI (1 µg/ml, Sigma, MO, USA) and mounted with Vectastain mountant (Vector Labs, CA, USA). The images were captured on Leica Stellaris Confocal Microscope (Leica, Hamburg, Germany). The hepatocytes were stained using ASGPR primary antibody tagged with Alexa fluor 594 (1:200, Cat. No. PA5-32030, Invitrogen, CA, USA).

### Western blot

Immunoblotting was performed to assess the difference in p21 protein expression among young, aged and HI tissues. Briefly, the tissue samples were first homogenized in the RIPA buffer added with protease and phosphatase inhibitor (Thermo Fisher Scientific, Waltham, MA USA), sonicated and clarified by centrifugation. The protein concentrations were determined by the DC (detergent compatible) protein assay (Bio-Rad Laboratories, Hercules, CA). Then, 40 µg protein was loaded onto a 12% SDS–PAGE, transferred onto polyvinylidene difluoride (PVDF) membrane and probed with p21 (1:200, Cat. No sc-6246, Santa Cruz Biotechnology, CA, USA) and actin (1:1000, Cat. No. ab 179467, Abcam) primary antibody and anti-mouse secondary antibody conjugated with horseradish peroxidase (1:10,000, Cat. No. 7076P2, Cell Signaling, Danvers, MA). The immune complexes were detected by chemiluminescence substrate (Thermo Fisher Scientific, Waltham, MA USA).

### Statistical analysis

Experiment appropriate statistical methods were used to analyze the results. The 2^ΔCt^ values for amplified senescence markers in tissues and doxorubicin-treated MEF cells were assessed using the Kruskal–Wallis test. The results of the H_2_O_2_-induced stress model were analyzed using a 2-way ANOVA (Uncorrected Fisher’s LSD), while p53 expression for H_2_O_2_-induced stress was analyzed using Wilcoxon’s and that of replicative senescence were analyzed by Mann–Whitney test. A *p*-value of <0.05 was considered as significant. The mRNA expression was assessed in seven mice/group with at least two technical replicates. The in vitro experiments were carried out in triplicates. The statistical analysis was performed using Graph Pad Prism software.

## Supplementary information

Supplementary material

## Data Availability

The corresponding author may be contacted to request the raw data and/or materials generated during this study.
