# Supplementary material for: p53 dependence of senescence markers p21v1 and p21v2 in aging and acute injury
Source: NPJ Aging. 2024 Oct 14;10(1):45. doi: 10.1038/s41514-024-00175-z (PMC11473800; doi:10.1038/s41514-024-00175-z)
Supplement: Supplementary file 1 — Supplementary material [file 41514_2024_175_MOESM1_ESM.pdf]

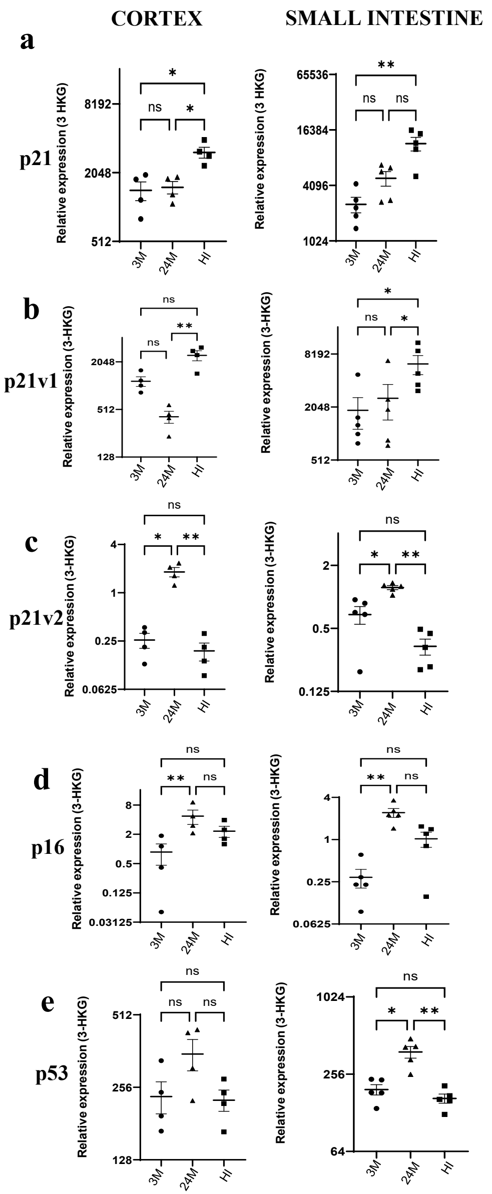

Supplementary Figure S1. mRNA expression of cdkn1a (p21), p21 variant1 , p21 variant 2, cdkn2a (p16) and p53 in cortex and small intestine (a-e) mRNA levels of p21 , p21variant 1, p21 variant 2 , p16 and p53 in the cortex and small intestine of male mice aged 3 months (3M, young) and 22-24 months (24M, aged) and male mice subjected to HI (HI, young mice). The data was normalized to geometric mean of three housekeeping genes (β-actin, β-glucuronidase and Rplp0 (ribosomal protein large P0)). The graphs are shown as individual data points along with mean ± SEM. The Y-axes are in $\log_{10}$ scales. Kruskal-Wallis non-parametric test was used. *p<0.05, **p<0.01, ***p<0.001, ns = not-significant. The Y-axes are in $\log_2$ scales.

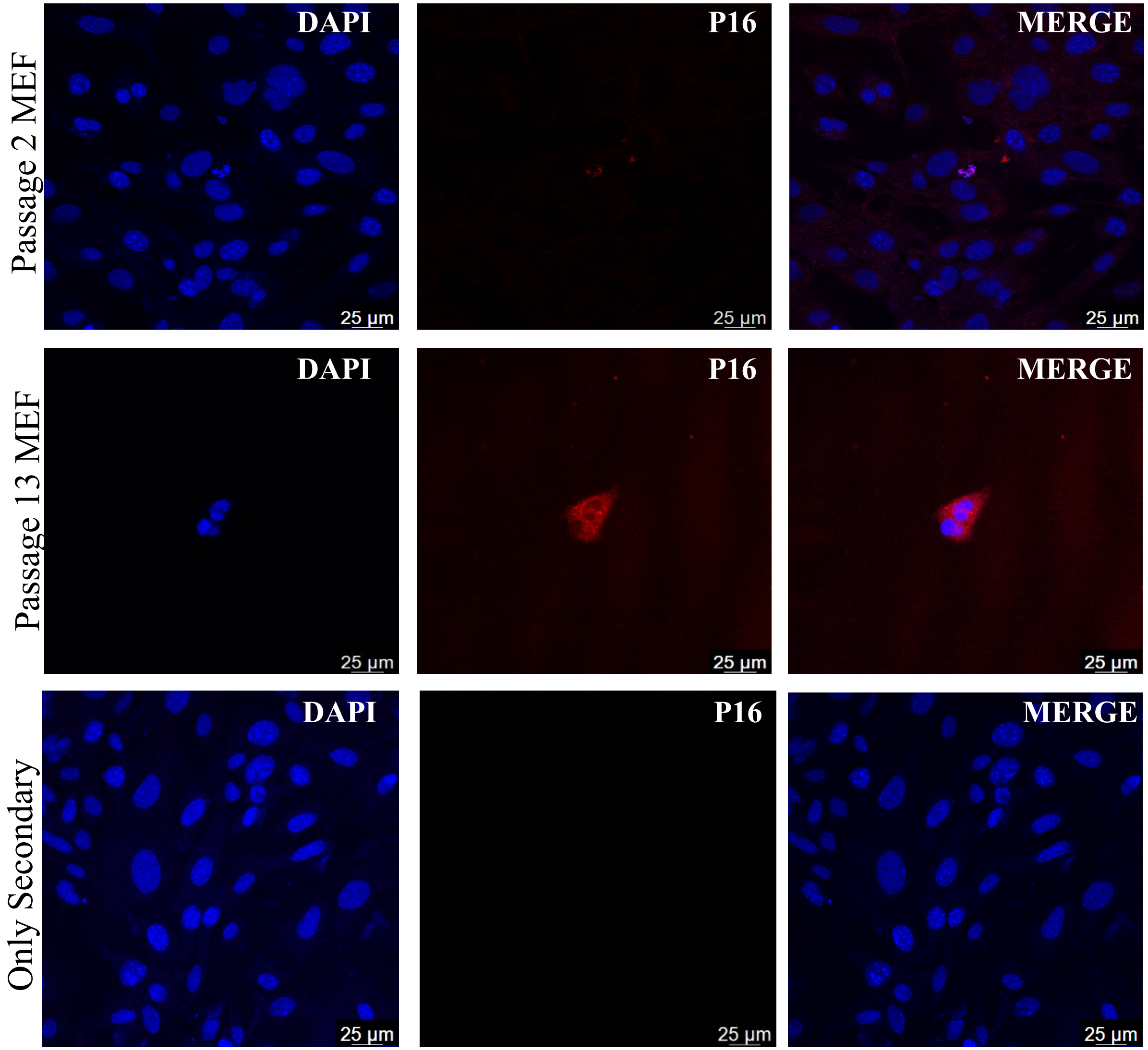

Supplementary Figure S2. Immunofluorescence image of p16 antibody staining in mouse embryonic fibroblasts (MEF) cells. The images shows initial passage and late passage MEF cells stained with p16 antibody. The late passage (P-13) cells showed high p16 staining when compared to initial passage (P-2) MEF cells, demonstrating the specific of p16 antibody. The nucleus is stained with DAPI. All the images were captured at a total magnification of 400X.

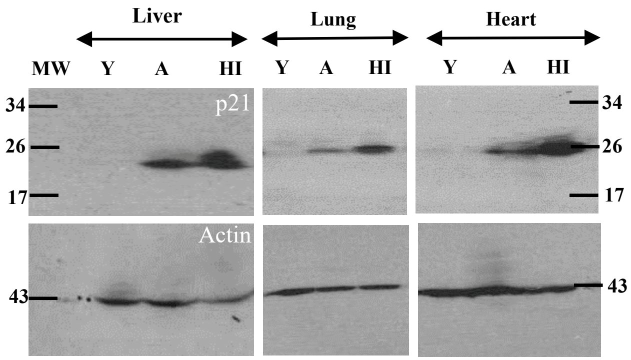

Supplementary Figure S3: Western blots showing the protein expression patterns of p21 in Liver , Lung and heart of young (3M) mice, aged mice and mice after HI. β-actin was used as internal control. 40μg protein was loaded in each well. MW – Molecular weight marker, Y- young mice (3M old), A-Aged mice (22-24M old), HI (mice subjected to hemorrhagic shock injury).

Supplementary Table S1. Mouse primer sequences used.

Gene	Forward primer sequence (5'-3')	Reverse primer sequence (5'-3')
p16ink4a/Cdkn2a	CCCAACGCCCCGAACT	GCAGAAGAGCTGCTACGTGAA
p21/Cdkn1a	GTTCCGCACAGGAGCAAAGT	ACGGCGCAACTGCTCAC
p21 variant 1	TCCACAGCGATATCCAGACA	GCACATCACCATTATTGGAC
p21 variant 2	TTGCCAGCAGAATAAAAGGTG	TTTGCTCCTGTGCGGAAC
p53	ATGGCCATCTACAAGAAGTCACAG	ATCGGAGCAGCGCTCATG
β-actin	CGCCACCACTTCGCCATGGA	TACAGCCCGGGGAGCATG
β-Glucuronidase	TCAACAACACACTGACCCCT	ACCCTTGGGATACATGGAGGT
Ribosomal protein large P0	AGATTCGGGATATGCTGTTGGC	TCGGGTCCTAGACCAGTGTTC
